# Microfluidic synthesis of methyl jasmonate-loaded PLGA nanocarriers as a new strategy to improve natural defenses in *Vitis vinifera*

**DOI:** 10.1038/s41598-019-54852-1

**Published:** 2019-12-04

**Authors:** Laura Chronopoulou, Livia Donati, Marco Bramosanti, Roberta Rosciani, Cleofe Palocci, Gabriella Pasqua, Alessio Valletta

**Affiliations:** 1 Chemistry Department, Sapienza Università di Roma, p.le Aldo Moro 5, 00185 Rome, Italy; 2 Environmental Biology Department, Sapienza Università di Roma, p.le Aldo Moro 5, 00185 Rome, Italy

**Keywords:** Microfluidics, Environmental monitoring

## Abstract

The objective of the present work was to synthesize biopolymeric nanoparticles (NPs) entrapping the resistance-inductor methyl jasmonate (MeJA) to be employed as a novel and alternative strategy in integrated pest management. NPs were prepared by using a continuous flow microfluidic reactor that allows to precisely control some features that are crucial for applications such as size, polydispersion, morphology and reproducibility. Poly(lactic-co-glycolic acid) (PLGA), a biopolymer largely studied for its use in biological applications, was chosen for the production of NPs entrapping MeJA, a biotic endogenous elicitor able to trigger plant’s defense responses. The effect of different fluid-dynamic conditions, PLGA molecular weight and concentration on NP properties (dimensions, polydispersion, morphology, stability) was evaluated. DLS and SEM were employed to characterize the obtained NPs. MeJA-loaded PLGA NPs ranging from 40 to 70 nm were administered to *Vitis vinifera* cell cultures, in order to evaluate the biological response in terms of stilbene biosynthesis. HPLC investigations showed a faster response when the elicitor was administered by PLGA NPs in comparison with free MeJA. This result demonstrates that the encapsulation in PLGA NPs significantly promotes MeJA cell uptake and the activation of MeJA-induced responses.

## Introduction

Nanotechnology is currently emerging in the sector of food science and crop production and it may offer new tools for developing more efficient and sustainable crop management techniques^1,2^. Biopolymeric nanoparticles (NPs) are widely used to deliver poorly water-soluble drugs, thanks to properties such as biocompatibility and biodegradability. The most common biopolymers used for nanobiotechnological applications include poly(lactic-co-glycolic acid) (PLGA), poly(ε-caprolactone), poly(cyanoacrylate), poly(ethylene glycol) as well as polysaccharides such as chitosan, dextran and hyaluronic acid. NPs design should ensure they possess specific properties that would allow to avoid repeated applications, i.e. stability, controlled and prolonged release and effective targeting^3^. An ideal system should also be able to penetrate the organism to reach the target site (pathogen or plant cells), resist the parasite/pathogen defenses, be biocompatible and eco-friendly and protect the cargo from atmospheric agents (preventing photodegradation, hydrolysis, leaching and volatilization). The use of biomaterials, that are generally biocompatible as well as biodegradable, ensures the concentrated bioactives are safer to handle by farmers.

Jasmonates are plant hormones involved in a multitude of biological mechanisms, including sprouting, root growth, geotropism, trichome, embryo and seedling development, leaf movement and senescence, fruit ripening and, above all, response to biotic and abiotic stresses^4^. These phytohormones, produced by most higher plants, are synthesized through the octadecanoid pathway from α-linolenic acid (α-LeA/18:3)^5^. Jasmonic acid and closely related methyl jasmonate (MeJA) are among the most thoroughly investigated. In the last years, many studies have been carried out on MeJA involvement in plant defences. As many works report, MeJA triggers the synthesis of antioxidant molecules and secondary metabolites^6^. One of the most important roles of jasmonates is their involvement in the response to the attack by different kinds of parasites (e.g. phytopathogenic fungi, bacteria, insects and nematodes). MeJA has been largely investigated as a resistance-inducer in different plant-model systems because of its engagement in the signal transduction cascade prompting defence responses. In tobacco and tomato, MeJA induces genes expressing pathogenesis-related (PR) proteins (i.e. chitinases, β-1,3-glucanases). Also, MeJA stimulates callose precipitation and the increase of PR-proteins in grapevine, inducing salicylic acid production^7,8^ and enhancing the biosynthesis of phytoalexins^9,10^. Periodic treatments of crops with elicitors, such as MeJA, could stimulate plants to produce phytoalexins, protecting them from pathogen attack, and thus significantly reducing the need of pesticide application. One of the main limitations to the use of MeJA both in the field and in post-harvest is its volatility which reduces its persistence. The entrapment of MeJA into nanocarriers capable of preventing its evaporation could be a promising strategy for the large-scale use of this elicitor.

*V*. *vinifera* L. lies among the most important crops, exploited worldwide and mainly for wine production. It is known that *Vitis* phytoalexins include a variety of polyphenols, among which stilbenes like trans-resveratrol are the most studied. These metabolites have shown a high toxicity against several pathogenic microorganisms such as *Botrytis cinerea*, *Erysiphe necator* and *Plasmopara viticola* (causative agents of grey mould, powdery mildew and downy mildew)^11^. On the other hand, in addition to being non-toxic for humans, they are considered precious nutraceutical compounds because of their beneficial effects on the human organism. The induction of stilbene biosynthesis in *V*. *vinifera*, while making the plant more resistant to pathogen attack, could also increase the health value of grape-derived products.

As it is well known, the plant cell wall represents the first point of interaction and a physical boundary for NPs uptake. The cell wall is semipermeable and possess pores whose dimensions determine its sieving properties. Thus, the pores present in the cell wall are likely to only allow NPs smaller than pore diameter to enter plant cells^12,13^. Current literature suggests that NPs properties such as chemical nature, shape, sizing and administration mode directly influence NP uptake, transport and accumulation in plant cells. Moreover, plant species is also relevant^13,14^.

Microfluidic devices have been designed to manipulate fluids in microchannels with the aim to accurately control the properties of synthesized NPs. Recently, we have described the use of a capillary microfluidic flow-focusing device for the fabrication of monodisperse biopolymeric NPs with diameters ranging between 35 and 350 nm^15,16^. In this work, we used this device to prepare MeJA-loaded PLGA NPs with dimensions suitable for their uptake by *V*. *vinifera* cells. The effect of free MeJA, in terms of stilbene biosynthesis, was correlated to that of MeJA-loaded PLGA NPs. The biosynthesis of the most common *Vitis* stilbenes in samples subjected to different treatments was evidenced through liquid chromatography.

## Results and Discussion

### Preparation of MeJA-loaded PLGA NPs

**Figure 1:**
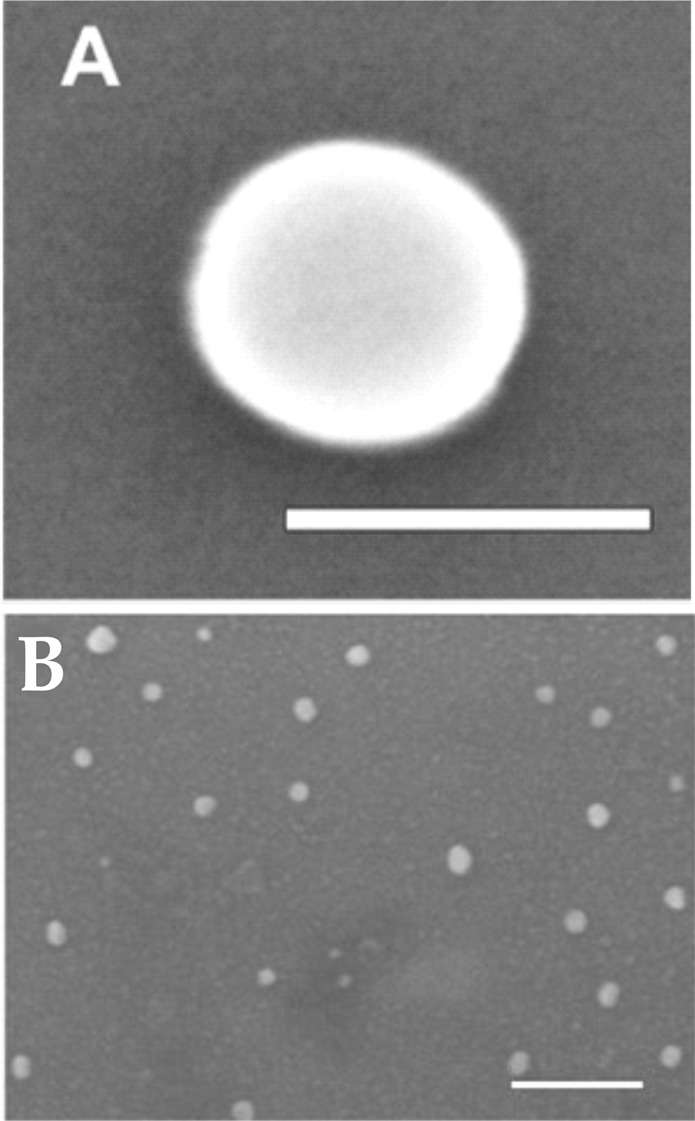
SEM micrographs of PLGA NPs. The dimension bars represent respectively 100 (**A**) and 200 nm (**B**).

We synthesized MeJA-loaded PLGA NPs with the microfluidic method described in the Methods section. According to our previous works, operating conditions (CH_3_CN as polymer solvent, capillary i.d. 254 μm and R = 0.05) were chosen to obtain NPs smaller than 50 nm^13^. In previous studies^13,17^ we observed that by using *V*. *vinifera* cell cultures, only NPs whose diameters were lower than 100 nm could penetrate through the cell wall and membrane and accumulate into the cytoplasm. In order to select the most suitable experimental parameters for the preparation of NPs smaller than 100 nm, we firstly carried out a study in which the initial concentration of polymer was varied by keeping constant the above-mentioned parameters. The drug/polymer weight ratio was kept constant and equal to 1:10. NP sizes, polydispersion indexes and zeta potentials are summarized in Table 1. Two representative SEM micrographs of the synthesized PLGA particles are reported in Fig. 1.

**Table 1:** Z-average diameters, polydispersity indexes (PDI), zeta potentials and loading % (w/w) of MeJA-loaded PLGA NPs synthesized with the microfluidic device working at different MeJA concentrations with a bioactive/polymer ratio of 1/10 (w/w).

MeJA* (mg/mL)	PLGA** (mg/mL)	NPs Diameter (nm)	PDI	Zeta potential (mV)	Loading % (w/w)
0.01	0.1	43 ± 21	0.254	−26 ± 11	4.6
0.05	0.5	52 ± 17	0.127	−31 ± 7	5.2
0.10	1	68 ± 22	0.112	−22 ± 8	5.4
0.20	2	73 ± 13	0.066	−34 ± 6	5.9
0.35	3.5	105 ± 36	0.132	−17 ± 9	5.2
0.50	5	124 ± 33	0.098	−27 ± 4	4.9

^*^Drug concentration in the DMSO organic phase.

^**^PLGA concentration in the DMSO organic phase.

The data reported in Table 1 clearly show that polymer concentrations greater than 2 mg/mL lead to NPs larger than 100 nm. As described previously^16^, higher polymer concentrations guarantee a good productivity and facilitate NP recovery from the outlet capillary device, therefore for all successive preparations we chose to work at a polymer concentration of 2 mg/mL.

**Figure 2:**
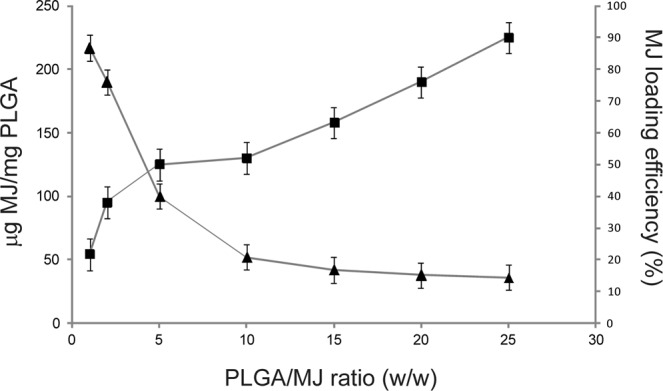
MeJA entrapment (triangles) and MeJA loading efficiency (squares) in PLGA NPs obtained by employing different PLGA/MeJA ratios (w/w) in the microfluidic synthesis.

The quantity of loaded MeJA per mg of NPs was derived from gas chromatography measurements, as described in the experimental section. Results are reported in Table 1. In the first set of experiments, the amount of entrapped MeJA did not seem to be related to PLGA concentration. After identifying the PLGA concentration of 2 mg/mL as the optimal one, we performed a series of experiments by keeping constant PLGA concentration and varying MeJA concentration in order to obtain different PLGA/MeJA ratios. MeJA loading (MeJA weight percentage in MeJA-loaded PLGA NPs) and the corresponding loading efficiency, defined as the percentage of MeJA entrapped with respect to the quantity used during NP synthesis, are shown in Fig. 2.

**Figure 3:**
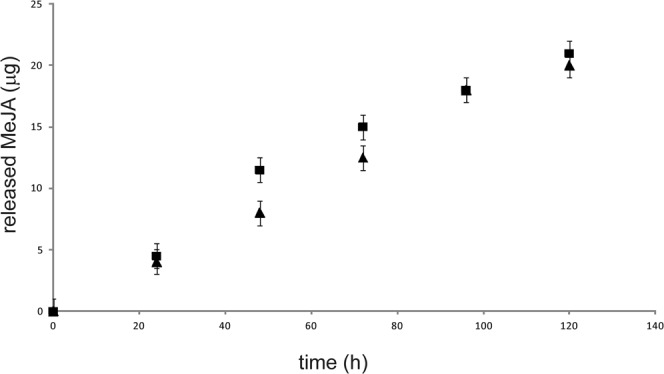
MeJA release kinetics from PLGA NPs in acetate buffer (pH 5.6) at 20 (triangles) and 30°C (squares).

We studied MeJA release kinetics from PLGA NPs in aqueous medium and at two different temperatures, 20 and 30°C, representative of the two main seasons in which plant treatment may be foreseen (spring and summer). It was not possible to determine the initial amounts of MeJA released at low incubation times (before 24 h) because it was below the sensitivity threshold of the instrument. MeJA release kinetics are reported in Fig. 3. The release of the bioactive substance occurs very slowly, and a plateau is reached after 120 h of incubation, corresponding to a concentration of released MeJA of 20 μg/mL. This trend can be explained by taking into account MeJA poor water solubility. The concentration of 20 μg/mL obtained at the plateau value is compatible with MeJA elicitation protocols on *V*. *vinifera* cell lines in which MeJA is commonly used at 25 μM (5.6 μg/mL). No relevant changes in MeJA release were observed at the two temperatures examined.

### Stilbene elicitation using MeJA-loaded PLGA NPs

The stilbenes production was analyzed in cell cultures of *V*. *vinifera* cv. Malvasia del Lazio subjected to elicitation with free MeJA or MeJA-loaded PLGA NPs. Cells were collected on days 6, 12, and 24 post-elicitation. The extracts were analyzed by HPLC to identify and quantify the four main *V*. *vinifera* stilbenes: *trans*-piceid, *trans*-resveratrol, *trans*-ε-viniferin and *trans*-δ-viniferin.

**Figure 4:**
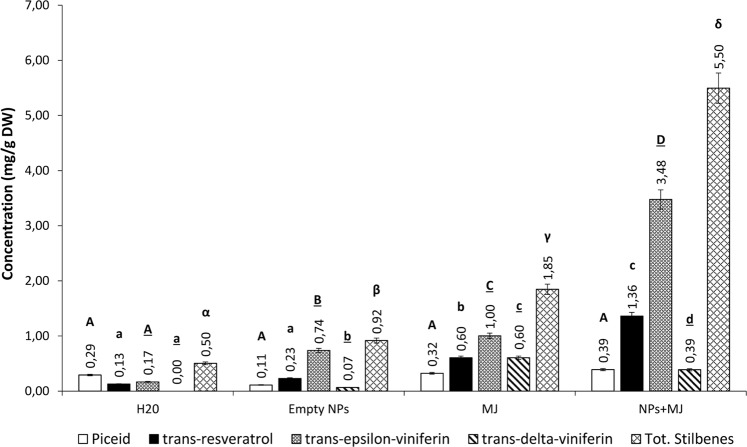
Stilbene concentrations in *V*. *vinifera* cv. Malvasia cell cultures subjected to different treatments and analyzed on day 6. Each value is the mean of 3 independent experiments ± S.D. Different letters indicate statistically significant differences (p ≤ 0.05) between samples. Comparison between the production under different treatments of: piceid (capital letters); resveratrol (lowercase letters); trans-ε-viniferin (underlined capital letters); trans-δ-viniferin (underlined lowercase letters); total stilbenes (Greek letters).

Six days after treatment, a relatively low total stilbene content (0.50 mg/g DW) was recorded in non-treated cells, while the treatment with empty PLGA NPs caused a doubling in stilbene concentration (0.92 mg/g DW) (Fig. 4). At the same experimental time, a 4-fold increase in total stilbene concentration (1.85 mg/g DW) was observed in cells treated with free MeJA. Cells treated with MeJA-loaded PLGA NPs showed an increase of total stilbenes production of about 11 times (5.50 mg/g DW) compared to the control and almost triple than free MeJA-treated cells. These increases were mainly due by *trans*-resveratrol and *trans*-ε-viniferin. In fact, *trans*-resveratrol concentration increased of about 4 times with free MeJA and of 10 times with MeJA-loaded PLGA NPs (from 0.13 to 0.6 and 1.36 mg/g DW, respectively). A 6-fold increase in *trans*-ε-viniferin content was detected in response to free MeJA and a 17-fold increase in response to MeJA-loaded PLGA NPs (from 0.17 to 1.00 and 3.48 mg/g DW, respectively). As far as *trans*-δ-viniferin is concerned, it was *de novo* synthesized in response to all treatments and the highest level (0.6 mg/g DW) was obtained with free MeJA. As for *trans*-piceid, different treatments did not cause significant differences in its detected levels.

**Figure 5:**
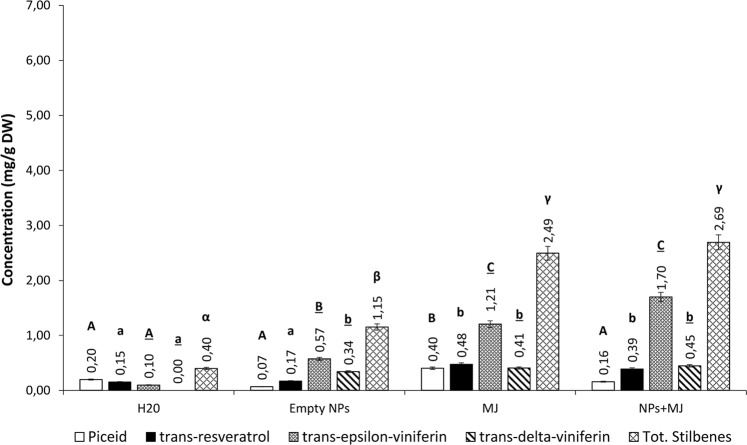
Stilbene concentrations in *V*. *vinifera* cv. Malvasia cell cultures subjected to different treatments and analysed on day 12. Each value is the mean of three independent experiments ± S.D. Different letters indicate statistically significant differences (p ≤ 0.05) between samples. Comparison between the production under different treatments of: piceid (capital letters); resveratrol (lowercase letters); trans-ε-viniferin (underlined capital letters); trans-δ-viniferin (underlined lowercase letters); total stilbenes (Greek letters).

At day 12 post-elicitation, the constitutive production of total stilbenes did not vary significantly in control cells (0.40 mg/g DW) (Fig. 5). In empty PLGA NPs-treated cells, a significant increase in stilbene concentration was observed (1.15 mg/g DW). Cell suspensions elicited with free MeJA showed an increase in total stilbene concentration of about 6 times (2.49 mg/g DW). By treating cells with MeJA-loaded PLGA NPs, an overall increase in total stilbene production of about 7 times (2.69 mg/g DW) was obtained. This increase was mainly due to *trans*-ε-viniferin, whose concentration increased of 12 and 17 times by using free MeJA and MeJA-loaded NPs (from 0.10 to 1.21 and 1.70 mg/g DW, respectively). However, 12 days after treatment the amount of total stilbenes was very similar in the case of free or entrapped MeJA cell elicitation.

**Figure 6:**
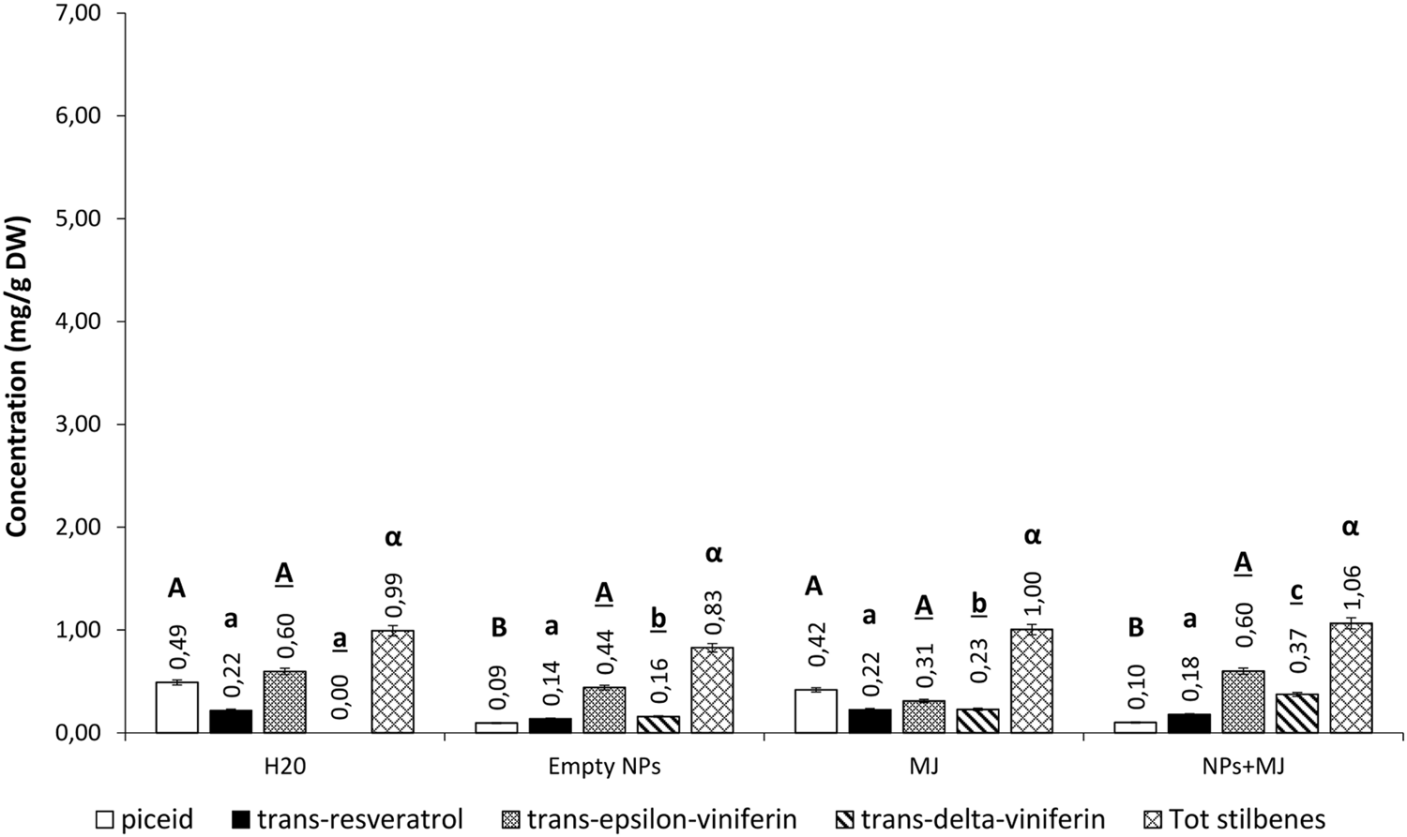
Stilbene concentrations in *V*. *vinifera* cv. Malvasia cell cultures subjected to different treatments and analysed on day 24. Each value is the mean of three independent experiments ± S.D. Different letters indicates statistically significant differences (p ≤ 0.05) between samples. Comparison between the production under different treatments of: piceid (capital letters); resveratrol (lowercase letters); trans-ε-viniferin (underlined capital letters); trans-δ-viniferin (underlined lowercase letters); total stilbenes (Greek letters).

At day 24 post-elicitation, the total stilbene content was 0.99 mg/g DW in non-treated cells. In cells treated with empty PLGA NPs, stilbene content remained almost constant (0.83 mg/g DW). A similar concentration was detected in cells treated with free MeJA (1.00 mg/g DW) and with MeJA-loaded PLGA NPs (1.06 mg/g DW) (Fig. 6).

The biocompatibility of PLGA has been extensively demonstrated in animal cells^18^, however detailed studies on plant cells are not currently available. We demonstrated that not only PLGA NPs are not cytotoxic for *V*. *vinifera* cells, as shown by the viability test (Fig. S1) but they also have an elicitor effect on stilbene biosynthesis. In particular, empty PLGA NPs stimulated the production of *trans*-ε-viniferin, one of the stilbenes with higher antimicrobial activity^19^. This implies that PLGA NPs may activate defence responses, independently from the cargo.

The most promising results were achieved on days 6 and 12 post-elicitation treatments. In fact, stilbene levels obtained in cell cultures treated with MeJA-loaded PLGA NPs were much higher than those observed with empty PLGA NPs or free MeJA, suggesting a synergistic effect between PLGA and the cargo of MeJA-loaded NPs.

When MeJA was delivered through PLGA NPs, it led to a faster cellular response in comparison to that obtained with the free elicitor. On day 6 after treatment, the total stilbene concentration was about 3 times higher than that obtained with free MeJA. This finding may be rationalized by considering the difficulty of MeJA to cross the plasma membrane. In fact, being PLGA a lipophilic polymer, it could facilitate MeJA cellular internalization through an endocytosis mechanism^13^ and reach faster the intracellular environment.

In agreement with literature data^20^, on day 12 after treatment with free MeJA stilbene accumulation peak occurred in *V*. *vinifera* cells. On day 12 post-elicitation, the effect of PLGA NPs on stilbenes production decreased, however it was still comparable to the effect obtained with the free elicitor. As a result, a single administration of MeJA-loaded PLGA NPs extended the exposure time of the cells to the elicitor by longer stimulating defence responses (from 6 to 12 days).

**Figure 7:**
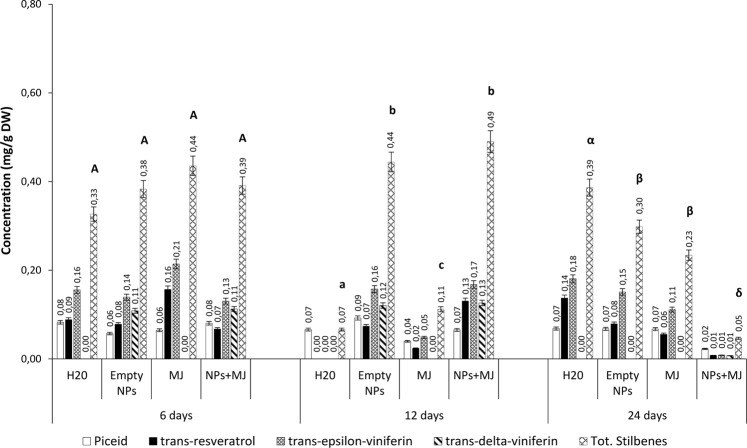
Stilbene concentrations released in the culture media after 6, 12 and 24 days after different elicitation treatments. Each value is the mean of three independent experiments ± S.D. Different letters indicates statistically significant differences (p ≤ 0.05) between samples. Comparison between the total stilbenes production at different time post elicitation: 6 days (capital letters); 12 days (lowercase letters); 24 days (Greek letters).

On day 24 post-treatment, a similar reduction in total stilbenes concentration was observed, both by using free MeJA (0.87 mg/g DW) and MeJA-loaded PLGA NPs (1.16 mg/g DW). The decrease in stilbene concentration could be explained by considering that stilbenes, being labile molecules with a high antioxidant activity, could undergo rapid oxidation^21^ and/or they could be released in the culture medium. However, in support of the first hypothesis, chemical analysis of culture media did not show a proportional increase in stilbenes concentration following both treatments at all considered times (Fig. 7). In the culture medium, the stilbene concentration was lower (0.49–0.05 mg/g DW) than in cell biomass. No differences in total stilbene concentration were observed on day 6 post-elicitation between the different treatments. On day 12 post-elicitation the highest stilbenes concentration in the culture medium was observed in response to empty PLGA NPs and MeJA-loaded PLGA NPs (0.44 and 0.49 mg/g DW, respectively). This could suggest that stilbenes release into the external environment is induced by the polymeric NPs and not by their cargo. On day 24 post-elicitation, the levels of stilbenes released in the culture medium decreased in all systems. Nonetheless, the maximum concentration was detected in the culture medium of non-treated cells as a consequence of a lower oxidative stress that corresponded to a lower stilbene degradation. Elseways, the minimal concentration was detected in the culture medium of cells treated with MeJA-loaded PLGA NPs which responded better in terms of stilbene production.

## Methods

### Materials

Poly(D,L)-(lactic-co-glycolic acid) (PLGA lactide:glycolide 50:50 MW 50 kDa), methyl jasmonate (MeJA) ≥95% in ethanol, acetonitrile (CH_3_CN) ≥99.9% for LC-MS and for HPLC, acetic acid, dimethyl sulfoxide (DMSO) ≥99.9%, acetone ≥99.5% for GC, ultrapure water for HPLC, coumarin 6 (Cu6) (98%), *trans*-resveratrol, *trans*-ε-viniferin standards and all other chemicals were from Sigma-Aldrich (Saint Louis, MO, USA) and used as received. Zero-Dead Volume Valco Internal cross-stainless steel 1/16″ OD × 0.25 mm Union bore, stainless steel capillary Tubing 1/16″ OD × 0.254 mm ID, were from Restek (Bellefonte, PA, USA). The samples of *V*. *vinifera* were given by the Istituto Sperimentale per l’Enologia (Velletri, Italy). Minisatr^®^ Syring Filters 0.2 and 0.45 µm were purchased from Sartorius (Gottingen, Germany).

### Microfluidic synthesis of MeJA-loaded PLGA NPs and drug loading evaluation

The microfluidic reactor employed in this study was previously described^15,16^. The flow rates of the continuous and dispersed phase were controlled by Carlo Erba SFC 3000 syringe pumps with a dedicated software (Maxima 820, Fisons Instruments). The microfluidic device was used to prepare MeJA-loaded PLGA NPs employing a flow-focusing configuration. PLGA (2 mg/mL) and MeJA (0.1–2 mg/mL) were dissolved in the organic phase (CH_3_CN) to obtain solutions with MeJA/PLGA weight ratios of 1: 25, 1:20, 1:15, 1:10, 1:5, 1:2 and 1:1. The solutions were infused in the central stream of the device. Ultrapure water was used as the continuous phase and infused in the side streams. All measurements were operated at least in triplicate and no precipitates were detected in the micro-channels during experiments. After sampling, the organic solvent was removed under reduced pressure. The NPs were centrifuged (18.600 × g, 20 min), washed and freeze-dried. The continuous phase flow rate was varied in the 400–5.000 μL/min range and the dispersed phase in the 20–600 μL/min range.

The quantitative analysis of MeJA was performed by a gas chromatographic method. We used an HP 6890 Gas chromatograph equipped with a Zebron ZB-WAX capillary column (30 m × 0.25 mm × 0.25 µm, fixed phase 100% PEG) and a flame ionization detector. The analytical determinations were obtained using the following instrumental set up: injection chamber temperature T_inj_ = 230 °C, column temperature T_col_ = 180 °C, detector temperature T_det_ = 280 °C, Split ratio 1/45. In such experimental conditions the retention time of MeJA was T_r_ = 7.42 min. Methyl mandelate (T_r_ = 4,23 min) was used as the internal standard. The calibration curve was obtained by preparing different concentration ratios between MeJA and the internal standard. For MeJA quantification, a known quantity of MeJA-loaded PLGA NPs was solubilized in 1 mL of chloroform. 1 mL of a methyl mandelate standard solution in chloroform (C = 2 × 10^−3^ M) was added to the obtained solution. The samples were then injected in the gas chromatographer and the resulting areas of the chromatographic peaks, corrected by the relative response factor, were compared with the calibration curve, allowing to calculate the amount of MeJA present in each sample and, consequently, MeJA loading in PLGA NPs.

### NPs sizing

The size and size distribution of the NP preparations were measured using a Nano Zetasize (Malvern Instruments, Malvern, UK). The experimental conditions were the following: a Helium-Neon laser operating at 633 nm, a fixed scatter angle of 173°, constant temperature T = 25 °C.

### Cell cultures and elicitation

*V*. *vinifera* cv. Malvasia (from Lazio region, Italy) cell cultures were obtained by inoculating 3 g fresh weight (FW) of calli in 100 cm^3^ flasks containing 20 mL of B5 liquid medium supplemented with 0.2 mg L^−1^ α-naphtaleneacetic acid (NAA), 1 mg/L kinetin (KIN) and 2% (w/v) sucrose^10^. The cultures were maintained under continuous darkness at 26 ± 1°C, on a rotatory shaker at 100 rpm. The suspended cells were elicited on day 10 of culture and collected on days 6, 12 and 24 after treatment. To evaluate the effectiveness of PLGA NPs in elicitor delivery, in terms of stilbenes production, *V*. *vinifera* cells were separately treated with MeJA 25 µM, administered in free form or entrapped in PLGA NPs. MeJA was solubilized in ethanol and diluted with sterile deionized water up to a concentration of 500 µM. To each flask,1 ml of solution was added to a final concentration of 25 µM. An equal volume of solvent (ethanol/water) was added both to control cells and to cells treated with empty-and MeJA-loaded-NPs.MeJA was added to the cell suspensions through filter sterilisation with Minisatr^®^ Syring Filters 0.2 µm. MeJA-loaded PLGA NPs suspension was added to the flasks prior sterilization with NaClO 2% for 30 min under continuous shaking. Control cells were treated with NaClO 2% and a second control was obtained by administering empty PLGA NPs. The cells were harvested by vacuum filtration, weighed and stored at 30°C until analysis^9^.

### Cell viability assay

The FDA viability assay was performed every 3 days during a culture period of 21 days on grapevine cell suspensions inoculated with empty PLGA NPs at a final concentration of 2.5, 5 and 15 mg/L, as described previously^9^. Cells were observed with a Zeiss microscope (Axioscop 2 Plus) using a Zeiss blue filter (λ_exc_ 386 nm; λ_emis_ 490 nm) and those that showed a green fluorescence in the nucleus and cytoplasm were regarded as vital.

### Stilbene extraction

On days 6, 12 and 24 post-elicitation, in order to study the intracellularly accumulated stilbenes, *V*. *vinifera* cells were subjected to three successive extractions (each 24 h) with MeOH/H_2_O (7:3 v/v) acidified at pH 3.0 with formic acid^22^. The final ratio between biomass and solvent was 1/10 (g FW/mL). A sonication of 20 minutes (30 mW/cm^3^; Elmasonic S 60/(H)) at a frequency of 37 kHz was carried out at the beginning of each extraction. To analyze the stilbenes released by cells in the culture media, a liquid-liquid extraction was performed. Ethyl acetate was chosen as the organic phase and the final ratio between organic and aqueous phase was 2:1 (v/v). The obtained extracts were dried under vacuum at 30 °C, redissolved in MeOH 70% with a ratio solvent/dry sample of 100/1 (µl/mg DW), and filtered through a 0.45 μm Minisatr^®^ filter^22^.

### Analytical determination of stilbene compounds

The analytical determination of stilbenes in the cell extracts was performed with an HPLC (Waters 1525 Instruments, Waters, Milford, MA) equipped with a dual wavelength detector (Dual λ Detector Waters 2487) and a C_18_ 4.6 mm × 100 mm Symmetry^®^ column with 5 µm porosity (Waters)^22^. The elution method was a multistep linear solvent gradient, going from 80% H_2_O to 50% H_2_O in 25 min; to 0% H_2_O in 2 min, plateauing for 6 min and to 80% in 2 min. Eluents were H_2_O (pH 3.2 with HCOOH) and CH_3_CN, both of HPLC grade. Stilbene quantification was performed by calibration curves obtained by analyzing *trans*-resveratrol (R^2^ = 0.997) and *trans*-ε-viniferin (R^2^ = 0.998) standards. Calibration curves were prepared by using six different concentrations of *trans*-resveratrol or *trans*-ε-viniferin from 0.006 to 0.2 mg/mL. The identification of piceid was based on the retention time of the corresponding peak of a *Polygonum cuspidatum* extract, whose chromatographic profile was previously characterized by HPLC/MS/DAD^22–24^. For piceid quantification, *trans*-resveratrol calibration curve was used. Identification of *trans*-δ-viniferin was carried out by comparison with the chromatographic profile of Vineatrol^®^, a commercial grapevine-shoot phenolic extract rich in *trans*-resveratrol, *trans*-ε-viniferin and *trans*-δ-viniferin, previously characterized through HPLC/MS/DAD^22,24^ and its quantification was performed on the *trans*-ε-viniferin calibration curve.

### Statistical analysis

Data significance among different samples for every time point was evaluated using the Student’s t-test. The differences were interpreted as significant when p ≤ 0.05.

## Conclusions

A microfluidic reactor was used to produce MeJA-loaded PLGA NPs by optimizing the effects of some key experimental conditions such as polymer concentration, microdevice geometry, solvent and flow rates with the aim to obtain controlled NPs size distributions. The delivery of MeJA, a biotic endogenous elicitor able to trigger plant defense responses, could be a novel and sustainable strategy for pest management. A significant amount of MeJA was entrapped in PLGA NPs, that were able to slowly release it in about 5 days. We evaluated the production of stilbenes by *V*. *vinifera* cells treated with free MeJA or MeJA-loaded PLGA NPs. The obtained results appear very promising for future applications as biopesticides to be released in crops. In fact, the use of PLGA NPs allowed to obtain a production of stilbenes that is earlier and more durable than free MeJA. Based on the results obtained, this system could be promising for future applications in the field to stimulate the natural defenses of plants.

## Supplementary information

Fi.S1

## Data Availability

The datasets generated during and/or analysed during the current study are available from the corresponding author on reasonable request.
