# Supplementary material for: Microfluidic synthesis of methyl jasmonate-loaded PLGA nanocarriers as a new strategy to improve natural defenses in Vitis vinifera
Source: Sci Rep. 2019 Dec 4;9:18322. doi: 10.1038/s41598-019-54852-1 (PMC6892798; doi:10.1038/s41598-019-54852-1)

Microfluidic synthesis of methyl jasmonate-loaded PLGA nanocarriers as a new strategy to improve natural defenses in *Vitis vinifera*

Laura Chronopoulou[a,§], Livia Donati[b,§], Marco Bramosanti[a], Roberta Rosciani[b], Cleofe Palocci[a,]*, Gabriella Pasqua[b] and Alessio Valletta[b]

[a]*Chemistry Department, Sapienza Università di Roma, p.le Aldo Moro 5, 00185 Rome, Italy*

[b]*Environmental Biology Department, Sapienza Università di Roma, p.le Aldo Moro 5, 00185 Rome, Italy*

Figure S1. Cell viability of V. vinifera cells treated with empty NPs evaluated through the FDA viability test. Cells observed after 6 days (A, B); after 12 days (C, D) and after 24 days (E, F). The scale bars represent 50 μm.

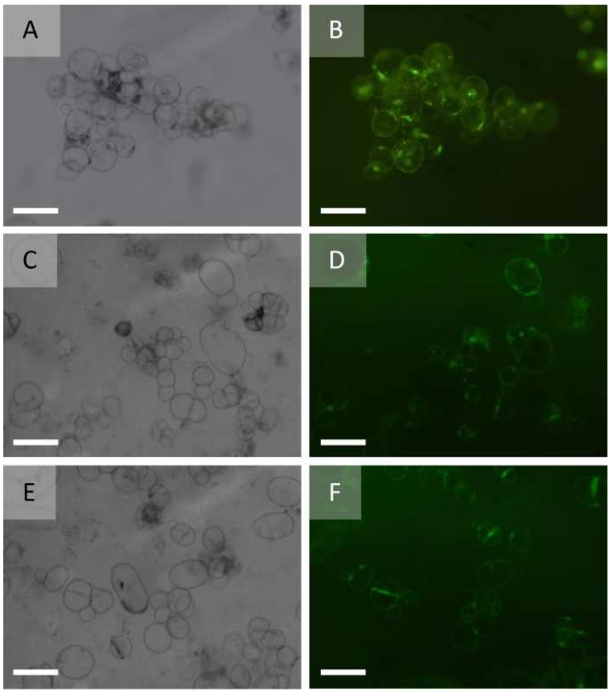

Supplement: Supplementary file 1 — Fi.S1 [file 41598_2019_54852_MOESM1_ESM.pdf]
